# Unraveling Homocysteine's Role in Dementia: No Specific Association with Alzheimer's Disease, but a Connection to White Matter Hyperintensities

**DOI:** 10.14336/AD.225.0020

**Published:** 2025-03-05

**Authors:** Théodore Decaix, Matthieu Lilamand, Karl Götze, François Mouton-Liger, Emmanuel Cognat, Jacques Hugon, Elodie Bouaziz-Amar, Louise Sindzingre, Julien Dumurgier, Claire Paquet

**Affiliations:** ^1^Geriatrics Department, Fernand Widal Lariboisière University Hospital, GHU APHP. Nord, Paris, France.; ^2^Paris-Cité University, Inserm U1144, Paris, France.; ^3^Geriatrics Department, Bichat University Hospital, GHU AP-HP. Nord, Paris, France.; ^4^Cognitive Neurology Center, Fernand Widal Lariboisière University Hospital, GHU APHP.Nord, Paris, France.; ^5^Biochemistry Department, Fernand Widal Lariboisière University Hospital, GHU APHP.Nord, Paris, France.; ^6^Paris-Cité University, Inserm U1153, Paris, France.

**Keywords:** Cognitive disorder, Vitamin B12, Hypertension, White matter hyperintensities, Clinical practice

## Abstract

Hyperhomocysteinemia (HHcy) is an established risk factor for cognitive impairment. The specific role of HHcy in the pathophysiology of Alzheimer's disease (AD) is debated, as most of the suspected mechanisms overlap with those of vascular dementia (VD). The aim of this study was to explore the association between plasma homocysteine (Hcy) levels and cerebrospinal fluid (CSF) biomarkers of AD, as well as brain magnetic resonance imaging (MRI) features. Cross-sectional observational analysis from a single-center tertiary memory clinic. We first assessed the association between Hcy in tertiles and the CSF AD biomarkers (according to the Amyloid (A) Tau (T) Neurodegeneration (N) classification) with further adjustments for age and sex. Then, we analyzed the relationship between HHcy and hippocampal atrophy (Scheltens scale) and white matter lesions (WML) (Fazekas scale) on brain MRI. We included 507 patients [mean age 68.9 (standard deviation=8.9)] with mean plasma Hcy at 13.3 (4.7) µmol/L in this study. There was no significant association between Hcy tertiles and CSF AD biomarkers. Plasma Hcy levels showed no correlation with any CSF AD biomarkers. The severity of WML increased with higher Hcy tertiles (*p*<0.0001). Patients with a CSF AD (A+T+) profile exhibited elevated mean Hcy levels when they presented moderate to severe WML (*p*<0.0001). Our findings challenge the link between Hcy and AD pathophysiology while highlighting a significant connection between Hcy and microvascular cognitive impairment. Further longitudinal studies are needed to validate these conclusions.

## INTRODUCTION

Alzheimer's disease (AD) is the most prevalent neurodegenerative disorder worldwide, accounting for a substantial share of the global dementia burden and highlighting the critical need for multifaceted therapeutic approaches, including prevention strategies. One of these targets is cardiovascular risk factors, as they are strongly associated with the risk of AD [1]. Managing cardiovascular health and associated risk factors is essential to mitigate the prevalence and economic impact of AD. Notably, hyperhomocysteinemia (HHcy), a recognized cardiovascular risk factor, has also been linked to an elevated risk of incident AD, along with other cardiovascular risk factors [2]. Homocysteine (Hcy), a marker related to vitamin B12 status, is associated with an increased risk of cognitive decline, accelerated cerebral atrophy, and the progression of white matter hyperintensities [3-5]. This modifiable risk factor can be effectively addressed through targeted B vitamin supplementation interventions [6].

HHcy is well known to cause brain microvessels alterations [7], with several proposed pathways contributing to HHcy-induced vascular dysfunction, including impaired endothelial function [8], oxidative stress [9], endoplasmic reticulum stress [10], inflammation [11], epigenetic modifications [12] and matrix metalloproteinase activation [13]. Preliminary evidence has suggested that the association between AD and HHcy may arise from the concomitant occurrence of AD and vascular dementia (VD) [14-16]. Moreover, the proposed HHcy-consequences-oxidative stress eventually leading to β-amyloid peptide production and cerebrovascular lesions-may be common to both HHcy-related AD and VD [17-20]. Vascular damage could therefore represent an underestimated confounding factor in the risk of HHcy-related AD.

Over the past decades, the use of AD-specific biomarkers, such as cerebrospinal fluid (CSF) β-amyloid and tau, has improved the diagnostic approach previously based solely on McKhann's clinical criteria [21]. These tools enable the *in vivo* identification of core AD lesions, leading to more precise and earlier diagnoses. As a result, amyloid and tau biomarkers have been incorporated into the latest revised definitions of AD.

The current body of evidence regarding the link between HHcy and AD does not rely on core amyloid and tau biomarkers [22], which are the cornerstone for AD diagnosis [23]. Only a limited number of studies have assessed the association between HHcy and CSF biomarkers of AD, and their findings have been inconsistent [24-28].

The objectives of this study were to examine the association between plasma Hcy levels and CSF biomarkers of AD, as well as brain magnetic resonance imaging (MRI) features in patients consulting for memory complaints in a tertiary memory clinic.

## METHODS

### Study design and participants

We conducted a cross-sectional single-center retrospective observational study at the Cognitive Neurological Center, part of the Tertiary Memory Clinic of Paris North, Assistance Publique-Hôpitaux de Paris, University Paris Cité, France, Lariboisière Hospital. The study took place between October 2009 and February 2021.

As previously published [29], patients experiencing memory complaints or other cognitive symptoms were referred to our clinic by their general practitioners or neurologists. All patients were evaluated by a multi-disciplinary team comprising neurologists, a geriatrician, neuropsychologists, a biochemist, a neuroradiologist, and a nuclear medicine physician, depending on the specific diagnostic requirements. This analysis focused on cognitively impaired patients with minor to major neurocognitive disorders, who underwent comprehensive clinical and biological assessments at our day-care department. This evaluation included CSF collection by lumbar puncture to measure amyloid beta (Aβ) 42 and 40, total-tau (T-tau), and phosphorylated-tau (P-tau) levels, in addition to assessing plasma Hcy levels. Plasma levels of vitamin B12 and folate were measured on the same day as Hcy. A brain magnetic resonance imaging (MRI) scan was also performed for all the patients as part of the routine assessment.

More precisely all patients that underwent lumbar puncture for AD biomarker assessment during the time of the study and for which Hcy plasma levels had been measured were included. Among them, those without a brain MRI were excluded.

### Biological and neuroimaging assessments

CSF amyloid and tau measurements were performed at Lariboisière Hospital's biochemistry unit using the Innotest® ELISA kits (Fujirebio, Gent, Belgium) until May 2018 and subsequently the ECLIA Elecsys® kits (Roche Diagnostics, Basel, Switzerland) for Aß42, Tau and P-Tau. According to the Amyloid Tau (Neurodegeneration) (also referred to as AT(N)) classification, patients were considered amyloid-positive (A+) if their CSF Aβ-42/Aβ-40 ratio or Aβ-42 level was below the established cut-off. They were considered tau-positive (T+) and neurodegeneration-positive (N+) if their P-Tau and total T-Tau levels exceeded the respective thresholds. Blood samples for Hcy analysis were collected by venipuncture between 8:00 and 9:00 AM after a 12-hour fast. Plasma samples were collected simultaneously with CSF and analyzed immediately without prior storage. Plasma Hcy levels, were measured by high-pressure liquid chromatography coupled to fluorimetric detection. It is important to note that clinicians were blinded to the Hcy plasma levels throughout the diagnostic process, and these measurements were considered only for research purposes.

Information related to neuroimaging data from the brain MRI, conducted outside the department, was collected by two physicians who were blind to the participants' clinical and biological data. Among these data, we analyzed hippocampal atrophy, which was assessed using the Scheltens scale, and the stage of white matter lesions (WML), measured with the Fazekas scale.

### Statistical analysis

Participants' characteristics were reported as means (standard deviation) for continuous variables and as frequencies and numbers for categorical variables. Initial univariate analyses compared characteristics across Hcy tertiles using ANOVA or Kruskal-Wallis tests for continuous variables, depending on their distribution, and the Chi-squared test for categorical variables. Then, linear regression models were performed to examine the relationship between plasma Hcy and the 3 AT(N) core CSF biomarkers, as well as the Scheltens and Fazekas stages. They were adjusted for clinically relevant variables. The Pearson correlation test was also used to assess the relationship between plasma Hcy and each CSF biomarker assayed up to May 2018. Student’s t-tests were performed to compare Hcy means according to the presence of cardiovascular risk factors. The mean Hcy levels were also compared according to the degree of hippocampal atrophy and WML in patients with an A+T+ CSF profile, indicative of biological AD. All statistical analyses and graphics were conducted using R software version 4.1.2, with a significance threshold set at a two-sided 5%.

### Ethical aspects

All participants received oral and written information about the opportunity to provide additional blood and CSF samples for further research under the BioCogBank© protocol. Written informed consent was obtained from all patients or their legal guardians, which included consent for the anonymous use of their clinical data and CSF results. The study was approved by the local Ethics Committee (Comité d’Evaluation et d’Ethique pour la Recherche Paris Nord, IRB 00006477 ref 16-004) and the Commission Nationale Informatique et Libertés.

**Figure 1.**
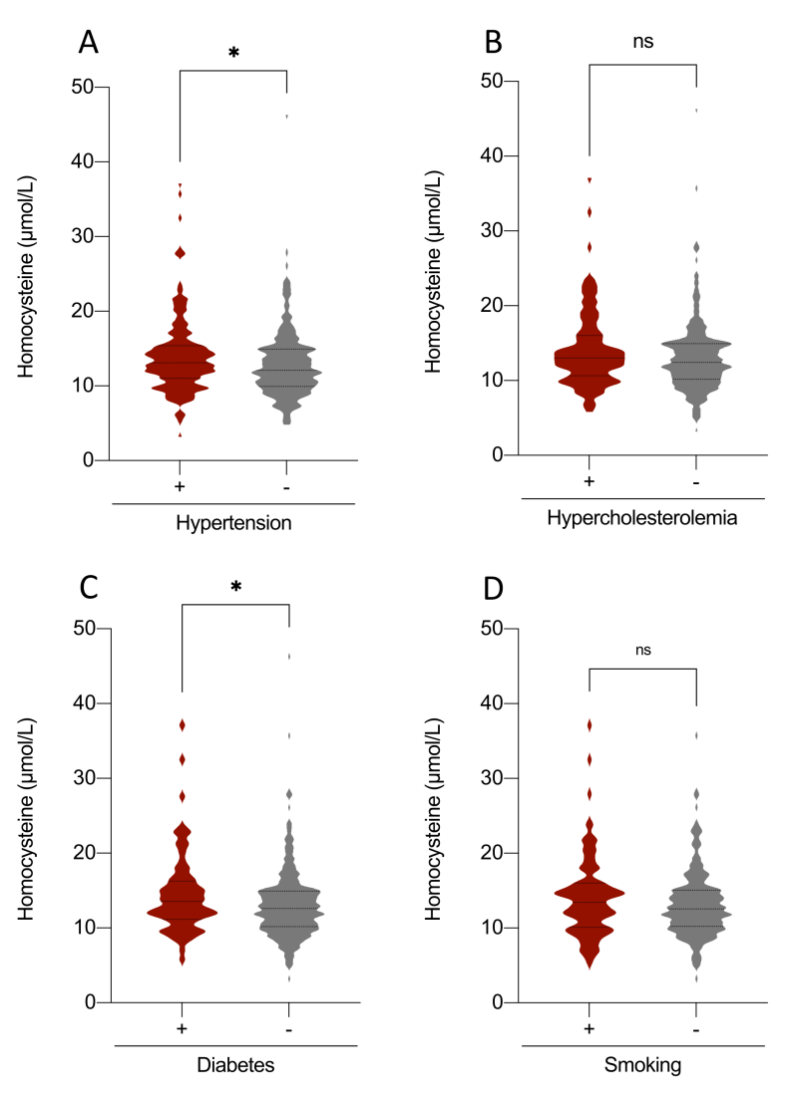
**Association between plasma homocysteine levels and cardiovascular risk factors**. Comparison of mean plasma homocysteine levels according to the presence or absence of four major cardiovascular risk factors: (A) hypertension, (B) hypercholesterolemia, (C) diabetes, and (D) smoking. Plasma homocysteine levels are presented as mean ± standard error of the mean (SEM). Statistical significance was assessed using a two-tailed t-test (n = 507). Significance thresholds: ns (not significant) > 0.05, * < 0.05, ** < 0.01, *** < 0.001, **** < 0.0001.

**Table 1:** Characteristics of the participants according to their plasma homocysteine level.

**Characteristics**	Total	Tertiles of homocysteine, µmol/L			p-value
	N = 507	< 11.20 N = 169	11.20 - 14.40 N = 173	> 14.40 N = 165	
**Age, years, mean (SD)**	68.9 (8.9)	66.7 (8.6)	69.7 (8.4)	70.2 (9.2)	<*0.001*
**Female, n, (%)**	266 (52.5)	113 (66.9)	82 (47.4)	71 (43.0)	<*0.0001*
**MMSE, mean (SD)**	22.8 (5.2)	22.9 (5.2)	23.0 (5.2)	22.5 (5.4)	0.5
**Level of education, n (%)**					
**Low (≤ 5 years)**	145 (28.6)	38 (22.5)	54 (31.2)	53 (32.1)	0.2
**Medium (6 - 8 years)**	167 (32.9)	63 (37.3)	57 (32.9)	47 (28.5)	
**High (≥ 9 years)**	187 (36.9)	67 39.6)	59 (34.1)	61 (37.0)	
**BMI, kg/m2, mean (SD)**	25.0 (4.7)	24.6 (5.1)	25.3 (4.9)	25.2 (4.2)	0.2
**Hypertension, n (%)**	204 (40.2)	55 (32.5)	77 (44.5)	72 (43.6)	0.1
**Hypercholesterolemia, n (%)**	134 (26.4)	39 (23.1)	53 (30.6)	42 (25.4)	0.5
**Diabetes, n (%)**	76 (15.0)	19 (11.2)	27 (15.6)	30 (18.2)	0.4
**Smokers, n (%)**	98 (26.6)	29 (23.6)	26 (21.1)	43 (35.0)	<*0.05*
***APOE* genotyping, n (%)**					
**ε3/ε3**	230 (45.4)	81 (47.9)	72 (41.6)	77 (46.7)	0.4
**ε3/ε4**	172 (33.9)	47 (27.8)	69 (39.9)	56 (33.9)	
**ε4/ε4**	35 (6.9)	15 (8.9)	10 (5.8)	10 (6.1)	
**ε2/ε3**	44 (8.7)	15 (8.9)	12 (6.9)	17 (10.3)	
**ε2/ε4**	6 (1.2)	2 (1.2)	1 (0.6)	3 (1.8)	
**ε2/ε2**	1 (0.2)	0 (0.0)	1 (0.6)	0 (0.0)	
**Folates, nmol/L, mean (SD)**	18.2 (8.3)	21.4 (8.8)	18.2 (7.4)	15.4 (7.7)	<*0.0001*
**Vitamin B12, pmol/L, mean (SD)**	338.0 (155.6)	367.1 (166.2)	328.2 (138.7)	320.3 (158.0)	*0.02*
**CSF biomarkers, pg/mL, mean (SD)**					
**CSF Aβ42**	864.1 (380.6)	896.0 (394.6)	870.5 (380.6)	825.0 (364.5)	0.1
**CSF Aβ40**	11564.3 (4529.4)	11840.1 (4537.1)	11656.5 (4504.3)	11181.6 (4551.2)	0.2
**CSF Aβ42/Aß40**	15.1 (8.2)	14.4 (7.0)	15.6 (8.4)	15.5 (9.0)	0.2
**CSF Tau**	372.7 (247.1)	373.7 (251.0)	362.0 (225.6)	382.8 (264.7)	0.7
**CSF p-Tau 181**	51.5 (34.6)	50.4 (32.4)	49.0 (33.3)	55.2 (37.8)	0.2
**MRI Characteristics**					
**Scheltens max**					
**0**	75 (14.8)	27 (16.0)	24 (13.9)	24 (14.5)	0.7
**1**	104 (20.5)	32 (18.9)	39 (22.5)	33 (20.0)	
**2**	166 (32.8)	55 (32.5)	60 (34.7)	51 (30.9)	
**3**	123 (24.3)	46 (27.2)	35 (20.2)	42 (25.4)	
**4**	38 (7.5)	8 (4.7)	14 (8.1)	16 (9.7)	
**Fazekas WML staging**					
**0**	123 (24.3)	58 (34.3)	41 (23.7)	24 (14.5)	<*0.0001*
**1**	191 (37.7)	77 (45.6)	60 (34.7)	54 (32.7)	
**2**	140 (27.7)	28 (16.6)	56 (32.4)	58 (35.1)	
**3**	52 (10.3)	7 (4.1)	15 (8.6)	30 (18.2)	

The data presented as mean (standard deviation) and number (percentage). Abbreviations: APOE: Apoliproteine E, BMI: body mass index, CSF: cerebrospinal fluid, MMSE: mini-mental state examination, MRI: magnetic resonance imaging WML white matter lesions.

## RESULTS

Of the 1,278 patients with biomarkers in the CSF during the study period, 742 had an Hcy measurement, among whom 507 had available MRI data. Therefore, the data of 507 patients were included in the present analysis. The mean age of participants was 68.9 (standard deviation = 8.9) years old, and 52.5% were females. The mean plasma Hcy level was 13.3 (4.7) µmol/L.

**Figure 2.**
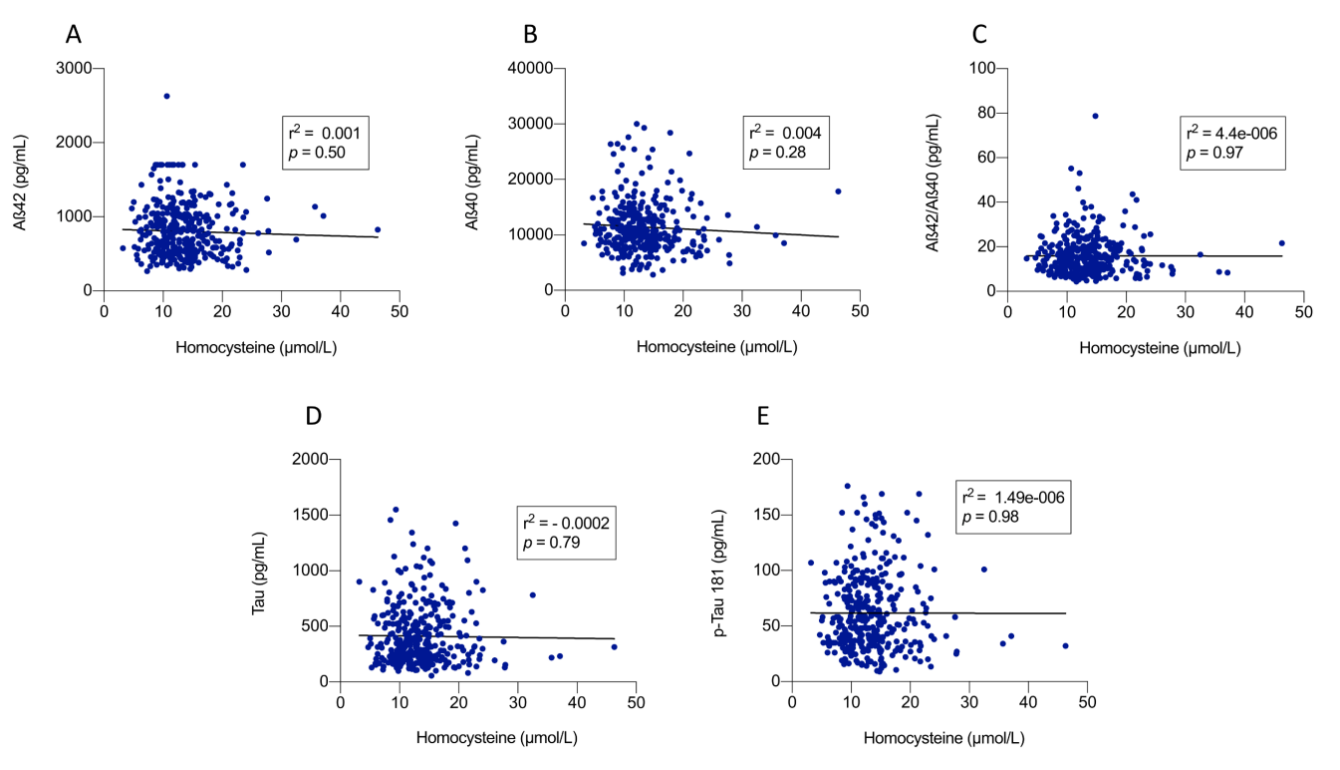
**Correlation between plasma homocysteine levels and Alzheimer's disease CSF biomarkers**. Scatter plots showing the correlations between plasma homocysteine levels and cerebrospinal fluid (CSF) biomarkers related to Alzheimer’s disease pathology: (A) Aβ42, (B) Aβ40, (C) Aβ42/Aβ40 ratio, (D) total Tau protein, and (E) phosphorylated Tau (p-Tau 181). Correlation coefficients were calculated using Pearson's correlation test (n = 355). Significance thresholds: ns (not significant) > 0.05, * < 0.05, ** < 0.01, *** < 0.001, **** < 0.0001.

The main characteristics of the study population are presented in Table 1. Individuals in the highest Hcy tertile were predominantly male (57.0%, *p* < 0.0001), smokers (35.0%, *p* = 0.05), with a mean age of 70.2 (9.2) years old (*p* < 0.001) and lowest mean plasma vitamin B12 (*p* = 0.02) and folates (*p* < 0.001). The mean MMSE of the total sample was 22.8 (5.2), with no significant difference between Hcy tertiles (*p* = 0.5). There was no significant association between Hcy tertiles and the mean levels of CSF biomarkers (Aß42, Aß40, T-Tau, P-Tau). A higher proportion of severe Fazekas grades (stage 3) was observed in the highest tertile (p < 0.0001), while the distribution of Scheltens atrophy stages did not show a significant difference (p = 0.7). Among cardiovascular risk factors, the mean Hcy levels were higher in patients with hypertension or diabetes [13.9 µmol/L (4.9) versus 12.9 µmol/L (4.5), *p* = 0.02 and 14.5 µmol/L (4.5) versus 13.1 µmol/L (4.5), *p* = 0.03, respectively] (Fig. 1). Hcy levels were higher in patients with higher stages of WML (Table 2). However, we did not find any association with hippocampal atrophy (Table 2). Linear regression analyses between AT(N) status and mean Hcy did not show any specific association with AT(N) patterns (Table 3). There was no significant correlation between plasma Hcy levels and CSF levels of Aß42, Aß40, T-Tau or p-Tau 181 (Fig. 2).

**Figure 3.**
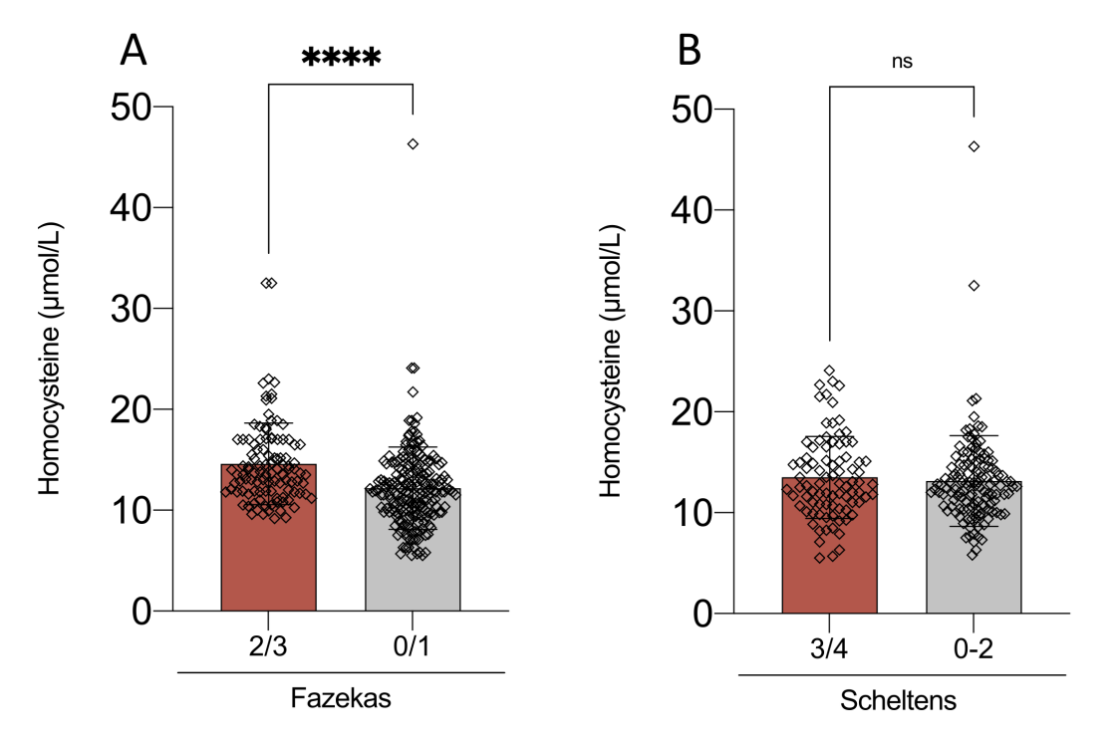
**Association between plasma homocysteine levels, white matter lesion severity, and hippocampal atrophy in A+T+ patients**. Comparison of mean plasma homocysteine levels according to (A) the stage of white matter lesions, assessed using the Fazekas scale, and (B) the degree of maximum hippocampal atrophy, evaluated with the Scheltens scale, in patients with a CSF A+T+ profile. Plasma homocysteine levels are presented as mean ± standard error of the mean (SEM). Statistical significance was assessed using a two-tailed t-test (n = 248). Significance thresholds: ns (not significant) > 0.05, *p < 0.05, **p < 0.01, ***p < 0.001.

Patients A+T+ with moderate to severe WML (Fazekas 2/3) had significantly higher mean Hcy levels [14.6 µmol/L (4.0)] compared to those with none to mild WML (Fazekas 0/1) [12.5 µmol/L (4.3) (p < 0.0001) (Fig. 3). Mean Hcy levels were significantly higher in A+T+ individuals presenting severe (Fazekas 3) WML vs Fazekas (0-2) [15.5 µmol/L (4.1) versus 13.2 µmol/L (4.3), *p* = 0.02] (data not shown). There was no difference in Hcy levels between subjects with higher stages of hippocampal atrophy (3-4) and those with no atrophy or mild atrophy (0 - 2) (*p* = 0.29) (Fig. 3).

**Table 2:** Relationship between levels of plasma homocysteine according to severity of white matter lesions and hippocampal atrophy.

	Mean (SD)	ß (SE)*	p-value*
**Homocysteine, µmol/L**			
**Fazekas 0**	11.5 (3.4)	Ref.	.
**Fazekas 1**	12.7 (4.9)	1.14 (0.52)	*0.02*
**Fazekas 2**	14.5 (4.3)	2.78 (0.56)	<*0.0001*
**Fazekas 3**	16.5 (5.5)	4.55 (0.76)	<*0.0001*
**Homocysteine, µmol/L**			
**Scheltens 0**	13.3 (4.8)	Ref.	.
**Scheltens 1**	12.7 (3.6)	- 0.68 (0.70)	0.3
**Scheltens 2**	13.4 (5.1)	- 0.47 (0.65)	0.5
**Scheltens 3**	13.3 (5.1)	- 0.85 (0.70	0.2
**Scheltens 4**	14.5 (4.2)	0.05 (0.95)	0.9

* adjusted for age and sex.

## DISCUSSION

This study examined Hcy concentrations in relation to core CSF biomarkers and brain MRI data in a real-life population of memory clinics patients, aiming to explore the pathophysiological link between Hcy, AD and VD. Hcy levels did not correlate with CSF amyloid or tau biomarkers, nor with the patients' AT(N) profiles i.e. diagnostic of AD according to current criteria [23]. Besides, there was no association with moderate to severe stages of hippocampal atrophy, typically suggestive of AD. Thus, there was no specific relationship between Hcy levels and markers of AD pathophysiological process and progression. In contrast, our study confirmed the association between Hcy levels and severe WML indicative of small vessel disease. Moreover, this association remained true in patients with biologically proven AD (i.e. A+T+ profile).

**Table 3:** Relationship between levels of plasma homocysteine and AD biomarkers CSF profiles.

AT(N) biomarkers profiles	Mean (SD)	ß (SE)*	p-value*
**Homocysteine, mmol/L**			
**A-T**-	13.5 (5.2)	Ref.	.
**A-T+**	12.4 (5.0)	- 0.98 (0.79)	0.2
**A+T**-	13.3 (4.3)	- 0.17 (0.72)	0.8
**A+T+**	13.3 (4.3)	- 0.64 (0.48)	0.2

* adjusted for age and sex.

CSF biomarkers are indicative of the neuropathological lesions associated with AD and enhance the accurate classification of AD patients in clinical studies [23]. In fact, the absence of core biomarker confirmation in clinical practice means that the risk of misdiagnosing AD is estimated to be between 20% and 30% [30]. Therefore, any assumptions regarding an association between HHcy and AD based solely on clinical definitions should be critically questioned, particularly given the lack of biomarker validation. This is why the aim of our study was to further investigate the potential link between HHcy and AD, utilizing appropriate biological tools for AD diagnosis confirmation. Indeed, research relying solely on clinical criteria for AD could produce misleading associations between AD and HHcy.

Our results are supported by those of a neuropathological study [31]. A post-mortem analysis of 265 patients revealed that the highest quartile of homocysteine (Hcy) levels was associated with neurofibrillary tangle count, though not with amyloid-ß load. The authors highlighted an association between Braak stage and the highest quartile of Hcy, which disappeared after adjusting for cardiovascular risk factors. Post-mortem brain MRIs were also conducted in this study, revealing an association between HHcy and periventricular WML. Nevertheless, the lack of a clear link with amyloid pathology calls into question the relationship between Hcy and AD.

Several observational studies in populations with similar age and sex distributions did not report any correlation between plasma Hcy levels and CSF biomarkers [24-28]. Popp et al. [24] included only 54 AD patients and 98 controls, reporting an association between p-Tau181 and Hcy-related metabolites, such as S-adenosylhomocysteine (SAH), 5-methyltetrahydrofolate (5-MTHF), and the SAM/SAH ratio in controls, as well as an association between p-Tau181 and SAH in AD patients. However, no association was found with Aβ42, one of the key hallmarks of AD pathology. The authors concluded that Hcy metabolism alterations are linked to neurofibrillary degeneration in both normal aging and AD, though this conclusion remains debatable as the absence of brain amyloid deposition generally rules out the hypothesis of AD. Alexopoulos et al. [25] analyzed a small sample of 88 patients with MCI or dementia, finding no correlation between Hcy levels and CSF biomarkers. Similarly, Smach et al. [26] studied 70 AD patients, 33 with other types of dementia, and 30 controls. While they reported differences in CSF folate levels between groups, with lower levels in AD patients compared to controls, no association was found between Hcy, CSF total Tau, and Aβ42. Dayon et al. [27] examined 120 older community-dwelling adults, incorporating Hcy and related metabolites in diagnostic models for cognitive decline but not specifically for AD. Hooshmand et al. [28], in a larger cohort of 462 patients (with CSF data available for 227), found an association between methylmalonic acid and lower Aβ42 levels but still no link with Hcy itself. None of these studies accounted for cardiovascular risk factors or WMH, which are major confounding factors for HHcy. Our study controlled these factors and analyzed data from a larger patient sample, further supporting the absence of a direct link between Hcy levels and CSF biomarkers with greater precision. In contrast, two studies reported an association between plasma Hcy and plasma levels of Aß [32, 33]. However, plasma measurement of Aß is still not validated for the diagnosis of AD to date. Moreover, cardiovascular risk factors were once again not considered in these studies.

Consistent with cardiovascular role of Hcy, hypertension and diabetes were associated with the highest plasma levels of Hcy in our study. Moreover, patients with large confluent WML indicative of small vessel disease also showed the highest plasma Hcy levels. In fact, cardiovascular risk factors, which are more prevalent with age, are implicated in the development of WML, which in turn may contribute to cognitive decline [34, 35]. AD is also associated with these risk factors, particularly hypertension [36 - 42]. In addition, HHcy is thought to increase the risk of hypertension by improving arterial stiffness and vasodilatation impairments [43, 44]. As it is closely associated with the same risk factors as AD, HHcy could be a confounding factor in cognitive decline due to cerebrovascular damage. Patients with VD may be inaccurately considered as affected by clinical AD, as long as there is no biomarker confirmation. Our approach challenges the hypothesis of a specific link between Hcy levels and AD by employing robust and well-validated biomarkers to assess biological features of the condition. Furthermore, patients with A+T+ CSF profile had increased Hcy levels in cases of severe WML, in our population. This supports the idea that Hcy may be related to the degree of vascular injury rather than to the diagnosis of AD itself. Small vessel disease is also a known factor in the progression of AD [45, 46]. Therefore, HHCy in A+T+ patients may appear as a pronostic factor, independent of AD itself.

Aging is one of the primary risk factors for dementia, both of neurodegenerative origin and due to vascular damage [47]. Vascular aging-related changes, including intracranial atherosclerosis, string vessels, capillary rarefaction, and blood-brain barrier dysfunction, contribute to neuronal loss and subsequent cognitive decline. Aging is also a key risk factor for cerebral amyloid angiopathy, a common comorbidity in AD that exacerbates the vascular burden of the condition. Hcy levels are also known to increase with aging, a finding confirmed by our results. Older patients often present with a combination of both neurodegenerative and vascular lesions, as they are exposed to shared risk factors for these pathologies, which may have cumulative or even synergistic effects [48]. This overlap makes it challenging to disentangle the respective contributions of age, AD pathology, and vascular damage to homocysteine levels. Therefore, we specifically investigated the relationship between Hcy levels, CSF biomarkers reflecting AD pathology, and WMH, systematically adjusting for age. While Hcy levels increase with aging, our findings suggest that elevated Hcy appears associated with the risk of developing VD without specific link to AD lesions.

The strengths of our study included the large number of patients enrolled, with AD diagnosis established using the most recent clinical-biological criteria. Furthermore, our data were derived from a real-world clinical practice cohort, which enhances the generalizability of the findings. However, our study has limitations that should be acknowledged. This monocentric study may be subject to selection bias, as it included only patients who underwent lumbar puncture. These cases likely represent diagnostic uncertainties, particularly when a neurodegenerative component was suspected despite the presence of significant vascular lesions. The absence of neuropathological confirmation of brain lesions does not allow for establishing a causal link between the pathologies and Hcy. MRI was performed on a large variety of equipments. MRI data were assessed by various neurologists and radiologists and were not exhaustively analyzed to account for all microvascular lesions. No information on the patients' nutritional status was provided, which could influence Hcy levels and introduce a potential bias in the classification of patients.

## Conclusion

This real-life study challenges the prevailing view of a direct link between Hcy levels and the biological signature of AD. Instead, it highlights a stronger association between Hcy levels and vascular cognitive impairment, primarily mediated by cardiovascular risk factors and white matter lesions. These findings suggest that Hcy may play a more pivotal role in vascular-related cognitive decline than in AD. To further clarify these relationships, longitudinal studies are needed to investigate the potential link between Hcy levels and the incidence of both AD and vascular dementia, which could provide a clearer understanding of its role in neurodegeneration.
